# Liver Injury Induced by Anticancer Chemotherapy and Radiation Therapy

**DOI:** 10.1155/2013/815105

**Published:** 2013-07-17

**Authors:** Y. Maor, S. Malnick

**Affiliations:** ^1^Department of Gastroenterology and Hepatology, Sheba Medical Center, 52621 Tel-Hashomer, Israel; ^2^Department of Internal Medicine C, Kaplan Medical Center, The Hebrew University of Jerusalem, 76100 Rehovot, Israel

## Abstract

Cytotoxic chemotherapy prolongs survival of patients with advanced and metastatic tumors. This is, however, a double-edged sword with many adverse effects. Since the liver has a rich blood supply and plays an active role in the metabolism of medications, it is not surprising that there can be hepatic injury related to chemotherapy. In addition, radioembolization may affect the parenchyma of normal and cirrhotic livers. We review chemotherapy-associated liver injury in patients with colorectal liver metastases, including downsizing chemotherapy and neoadjuvant chemotherapy. We discuss the mechanism of the hepatic injury, secondary to reactive oxygen species, and the spectrum of hepatic injury including, steatosis, steatohepatitis, hepatic sinusoidal injury and highlight the pharmacogenomics of such liver insults. Methods for reducing and treating the hepatotoxicity are discussed for specific agents including tamxifen and the newly introduced targeted antibodies.

## 1. Introduction 

Over the last few decades many novel cytotoxic chemotherapeutic agents have been developed which prolong survival of patients with advanced and metastatic tumors., More recently, specifically targeted antibodies and other biological agents have been introduced in various combinations with chemotherapy to further increase life expectancy. For some tumors, for example, colorectal cancer (CRC), preoperative treatment may “downsize” liver metastases to make them compatible with complete resection with a curative intent. External radiation therapy has been an integral part of the armamentarium against primary or metastatic liver tumors. Currently, radiation may be directly targeted at liver tumors with the radioembolization technique. This increased availability of beneficial treatment modalities does not come without a price. Administration of chemotherapy has always been complicated with many adverse effects. In this review we will focus on the effects of chemotherapy and radiotherapy on the liver. The liver may be affected by various pathological manifestations, some culminating in severe liver injury and even liver failure. Chemotherapy-induced liver injury may also bear on the morbidity and mortality after hepatic resection. Radioembolization, although relatively safe, may affect the parenchyma of normal and cirrhotic livers. 

## 2. Chemotherapy-Associated Liver Injury in Patients with Colorectal Liver Metastases

In the absence of any treatment, the prognosis of patients with liver metastases from CRC is dismal [1]. In those patients with resectable disease, liver surgery with complete resection of the metastases has markedly improved long-term survival [2]. The most significant advance regarding CRC over the past decade has been the introduction of several effective cytotoxic chemotherapeutic agents mainly 5-Fluorouracil (5-FU), oxaliplatin, and irinotecan [3]. Further benefits were achieved by the addition of monoclonal antibodies directed against epidermal growth factor receptor (EGFR) or against vascular endothelial growth factor (VEGF), for example, bevacizumab [4]. In patients with metastatic CRC treated in a palliative intention, the combination of oxaliplatin- or irinotecan-based chemotherapy with an antibody increased the median overall survival from 20 to 22 months [4]. Advances in systemic therapy for metastatic CRC have led to more patients being treated with chemotherapy before hepatic resection. For patients with initially unresectable metastases, preoperative therapy can lead to a decrease in the size of metastases and render these patients resectable—referred to as “downsizing chemotherapy” [5, 6]. For patients with initially resectable metastases, progression free survival improves with perioperative chemotherapy compared with surgery alone—this is termed “neoadjuvant chemotherapy.” There is less evidence, however, on the beneficial effect of neoadjuvant chemotherapy alone on survival [7]. Potential disadvantages of preoperative chemotherapy are the risk of disease progression before surgery and liver toxicity. Chemotherapy induces various histological changes of the liver parenchyma including steatosis, chemotherapy-associated steatohepatitis (CASH), or sinusoidal injury sinusoidal obstruction syndrome (SOS) [8–10]. Agreement exists on a link between the chemotherapy-associated changes and poor postoperative outcomes. Hepatic parenchymal injury is regimen specific. For example, irinotecan-based regimens are associated with steatohepatitis (number needed to harm 12; 95% CI 7.8–26) whereas oxaliplatin-based regimens being can result in grade 2 or greater sinusoidal injury (number needed to harm 8; 95% confidence interval [CI] 6.4–13.6) [11]. 

## 3. Mechanism 

The mechanism of chemotherapy-induced hepatic injury is thought to be secondary to production of reactive oxygen species (ROS), intended to induce tumor cell apoptosis [12]. Previously steatotic livers were thought to be most susceptible to chemotherapy-induced injury due to impaired regenerative capability and abnormal innate immunity [13–15]. 

## 4. Clinical-Pathological Modes of Liver Injury

### 4.1. Nonalcoholic Fatty Liver Disease

The epidemic of obesity, insulin resistance, and the resulting metabolic syndrome has led to an increased prevalence of nonalcoholic fatty liver disease. This has been estimated to be present in more than 20% of patients planned for hepatectomy [16]. Steatotic liver is more vulnerable to injury from general anesthesia and ischemia/reperfusion [17]. Protective mechanisms against oxidative stress are significantly impaired in steatosis [18], and impaired energy homeostasis further sensitizes steatotic livers to surgical stress [16]. Regeneration is delayed in steatotic livers [16, 19], with a resulting prolongation of liver dysfunction [16]. 

### 4.2. Steatosis

The effect of mild to moderate steatosis without associated inflammation on postoperative outcome is likely to be small. Kooby and colleagues [20] found that, in patients with steatosis who underwent major liver resection, steatosis was associated with infection-related complications but not with major complications or postoperative mortality. However, many patients with steatosis have other comorbid conditions, such as obesity and diabetes that can increase the risk of complications. In a study of patients who had major hepatectomy, patients with steatosis had increased blood loss, more postoperative complications, and a longer mean intensive-care-unit stay per patient as compared with matched control patients with healthy livers [21]. Fluorouracil (5-FU), which remains the backbone of modern chemotherapy, has been linked to the development of steatosis. Reports indicate the development of steatosis in 30 to 47% of patients after 5-FU therapy, although some changes may be reversible [22–24]. 

### 4.3. Steatohepatitis

Irinotecan is clearly associated with steatohepatitis, with a rate of 20.2% seen in patients administered this drug, compared with 4.4% in those not having chemotherapy [25–27]. This effect was exacerbated by baseline obesity [28]. Steatohepatitis was found in 24.6% in those with a BMI of 25 kg/m^2^ or more who were administered irinotecan, but only 12.1% in irinotecan treated patients with a BMI less than 25 kg/m^2^ [26]. Steatohepatitis increases the risk of liver failure [28, 29] and postoperative complications [18] following major hepatectomy. Primarily because of its effect on regeneration, steatohepatitis is also associated with increased overall postoperative mortality [26]. An almost 10-fold-increased 90-day mortality following hepatectomy in patients with steatohepatitis (mortality 14.7% versus 1.6%) with a six-fold higher risk of death from postoperative liver failure (5.8% versus 0.8%) has been reported [26]. Major hepatic resection should probably be avoided in patients with known steatohepatitis, as should irinotecan in patients with known steatosis or steatohepatitis in whom major hepatic resection is planned. 

### 4.4. Hepatic Sinusoidal Injury

Sinusoidal injury ranges from sinusoidal dilation to hepatic sinusoidal obstruction syndrome, also termed venoocclusive disease, which can progress to regenerative nodular hyperplasia [30]. Injury to the sinusoidal endothelial cells lining the sinusoids, the initial event, leads to subintimal thickening and extravasation of erythrocytes into the subendothelial space of Disse (perisinusoidal space). Sinusoidal endothelial cells and erythrocytes embolize in sinusoids and block venous outflow, resulting in hepatic congestion and sinusoidal dilatation. At later stages, a fibrotic reaction in the sinusoids can lead to obliteration of central venules, leading to hepatic sinusoidal obstruction syndrome. Rates of injury are universally higher in patients receiving oxaliplatin-based chemotherapy. Oxaliplatin was associated with a 10-fold increase in sinusoidal dilation compared with no chemotherapy (18.9% versus 1.9%) [26]. In the European Organisation for Research and Treatment of Cancer EORTC-4098336 study involving administration of chemotherapy, high-grade injuries were much more prominent in the group administered chemotherapy (41%) compared to the control group (0%) [31]. Sinusoidal changes may normalize with time after cessation of chemotherapy, and delaying surgery for several months might be a useful option in patients with diagnosed or suspected sinusoidal injury [32]. Oxaliplatin and other platinum compounds lead to the generation of ROS and could result in depletion of glutathione from sinusoidal endothelial cells (SECs) [33, 34]. Cisplatin has been shown to cause actin dissociation, which can upregulate matrix-metalloproteinases-9 (MMP-9) activity [35]. Morbidity following hepatectomy is significantly higher in patients with evidence of sinusoidal obstruction syndrome, although there is no increase in mortality [36]. Sinusoidal injury also significantly increases hospital stay [32]. Oxaliplatin is associated with an increased transfusion requirement compared to patients receiving 5 FU/leucovorin or no chemotherapy [37]. It has been suggested that increased blood loss is directly attributable to these vascular lesions. Transfusion is associated with an adverse outcome after hepatectomy and a requirement for red cell transfusion is independently associated with overall morbidity [38]. Mortality was increased from 1.2%, when transfused 2 units of blood or less following hepatectomy, to 11.1% for those requiring more than 2 units [38]. 

## 5. Pharmacogenomics

It is increasingly recognized that pharmacogenomics can play a key role in determining the susceptibility of the individual to the toxic effects of chemotherapy. Recently, a randomised trial showed that patients given an irinotecan dose modified on the basis of CYP3A enzymatic activity had reduced interindividual pharmacokinetic variability for irinotecan and its active metabolite, SN-38, and a decreased incidence of severe neutropenia [39]. SN-38 is also inactivated by glucuronidation, which is metabolised by the UGT1A1 enzyme. A polymorphism in the promoter of the gene encoding UGT1A1 results in lower rates of SN-38 glucuronidation, leading to worse diarrhea and neutropenia associated with irinotecan [40]. Similarly, oxaliplatin toxicity is affected by mutations in genes involved in DNA damage repair and conjugation of its metabolites to glutathione [41]. 

## 6. Diagnosis

CT is the most widely used imaging technique to investigate CRC liver metastases and is useful for detecting moderate to severe steatosis (hepatic fat content of more than 30%) [42]. MRI was more accurate than noncontrast CT in the diagnosis of steatosis, particularly in patients with BMI of 30 kg/m^2^ or more [43]. However, available imaging techniques cannot differentiate steatohepatitis from steatosis or identify sinusoidal injury. Biopsy is the definitive method for the diagnosis of chemotherapy-induced liver injury. Some investigators have advocated staging laparoscopy to visually inspect and sample liver parenchyma prior to performing hepatic resection [8]. This approach, however, may be difficult to apply in routine clinical practice. 

## 7. Prevention and Treatment

Postoperative morbidity was related to the duration of preoperative chemotherapy, with higher morbidity rates among patients who received at least six cycles or more compared with those who received fewer than six cycles [36]. The optimum duration of preoperative chemotherapy, to maximize therapeutic benefit while avoiding hepatotoxicity, is likely to be 4 months [26]. Several studies show that a longer interval between chemotherapy and hepatic resection reduces hepatotoxicity and surgical complications. However, this interval should be balanced with the risk of tumor progression during the treatment-free interval. An interval of 5 weeks is recommended to minimize postoperative complications while avoiding a long delay in treatment [44, 45]. Patients with suspected chemotherapy-associated liver injury who need major hepatic resection should have assessment of their functional future liver remnant to minimize postoperative complications. Methods to predict the function of the expected remnant liver include biochemical tests for hepatic clearance of compounds, such as indocyanine green, and measurement of the future liver remnant measured with CT. In patients with normal liver function, a minimum of 20% is needed to prevent complications after major hepatectomy. In patients with substantial chemotherapy-induced liver damage, a future liver remnant of 30% has been proposed as the minimum volume needed before major hepatic resection. When the future liver remnant is predicted to be insufficient for safe hepatic resection, portal-vein embolization of the part of the liver to be resected can induce hypertrophy of the future liver remnant. Liver remnant hypertrophy of less than 5% is associated with increased postoperative morbidity [46]. Patients with substantial chemotherapy-associated liver injury who have inadequate liver hypertrophy after a portal-vein embolization are not candidates for a major liver resection. Hepatic pedicle clamping (Pringle manoeuvre) to limit blood loss is an integral component of major hepatectomies but may deliver an ischaemic/reperfusion injury to the remnant liver [47]. Adding the anti-VEGF antibody bevacizumab may have a protective effect against oxaliplatin-induced sinusoidal injury. Bevacizumab lowered the incidence of sinusoidal dilatation in patients receiving oxaliplatin and become reduced to less than a third that of severe (grade 2-3) sinusoidal dilatation (8 versus 28%) [48]. However, the overall complication rate was not significantly different between those who received oxaliplatin-based neoadjuvant chemotherapy with or without bevacizumab [49]. 

## 8. Tamoxifen-Induced Nonalcoholic Steatohepatitis and Injury Inflicted by Other Hormonal Agents

Tamoxifen is an estrogen-receptor antagonist and at a dose of 20 mg/day is the adjuvant hormonal treatment of choice in women with estrogen-receptor-positive breast cancer [50]. Severe side effects are unusual with tamoxifen, but it is associated with the development of nonalcoholic fatty liver disease (NAFLD) and non alcoholic steatohepatitis (NASH) [50]. NASH is the most prevalent form of progressive liver disease and it is suggested that NAFLD affects 10–39% of the global population. Drugs account for less than 2% of the causes of NASH. Drugs known to be capable of inducing steatosis and steatohepatitis can be divided into three broad groups: those that cause steatosis and steatohepatitis independently (e.g., amiodarone, perhexiline maleate); drugs that can precipitate latent NASH (e.g., tamoxifen); and drugs that induce sporadic events of steatosis/steatohepatitis (e.g., carbamazepine) [51]. An Italian multicentre trial [52] enrolled 5408 healthy women who had hysterectomies, in which half of the women were prospectively assigned to tamoxifen 20 mg daily and the other half to placebo for 5 years. This study showed that the major risk factors for the development of tamoxifen-induced NASH were obesity (central), hyperlipidaemia, and diabetes. The incidence of tamoxifen-induced NAFLD was estimated to be about 40% at 1 year. Overall, tamoxifen was associated with an increased risk of developing NAFLD (hazard ratio: 2.0, 95% confidence interval [CI]: 1.1–3.5), but the association was restricted to overweight women. The increased risk was limited to the first two years of treatment. Mild to moderate non-alcoholic steatohepatitis, when recognized at the onset, seemed to be indolent in the long term, and no progression to cirrhosis was observed after a median followup of 8.7 years [52]. In a large registry of 1105 patients with breast cancer [53], NASH was documented in 2.2%. Seventeen patients (1.5%) developed NASH after their diagnosis of breast cancer. Multivariate logistic regression analysis indicated that age, BMI, and tamoxifen were significant factors associated with NASH. The odds of developing NASH increased 8.2-fold when patients were treated with tamoxifen. The median time from the start of tamoxifen to documented NASH was 22 months. NASH improved after tamoxifen was stopped. Only 2 patients had biopsy-documented cirrhosis in 806 patients who took tamoxifen [53]. Therefore, cirrhosis is considered a rare complication of tamoxifen therapy. Tamoxifen was shown to increase hepatic fat content, through blocking the role of estrogen in maintaining hepatic lipid homeostasis by supporting the expression of genes involved in lipid *β*-oxidation. In addition, tamoxifen has been shown to increase serum triglyceride and lower low-density lipoprotein and cholesterol levels. It was suggested that fatty liver is vulnerable to oxidants and progresses to steatohepatitis when a second agent (such as tamoxifen) generates liver cell death, inflammation, and activation of stellate cells with production of fibrosis (multiple hit hypothesis) [54, 55]. Increased TNF-*α*, mitochondrial *β*-oxidation rates and the production of large amounts of reactive oxygen species play a major role in drug-induced steatohepatitis [56]. Interestingly, the difference in the distribution of CYP genotypes between patients may increase the individual susceptibility for tamoxifen-induced NASH [57]. In addition, serum leptin levels were found to be significantly elevated in patients with hepatic steatosis after tamoxifen treatment [58]. Risk factor management is the most important step in the treatment of NASH. In case of severe NASH, tamoxifen should be stopped and one of the aromatase inhibitors can be started. 

Anastrozole is a selective aromatase inhibitor approved for the treatment of postmenopausal hormone-sensitive breast cancer. Few cases of acute hepatitis occurring during treatment with anastrozole have been reported [59, 60]. In one report [60] liver biopsy revealed diffuse liver cell necrosis in acinar zone 3, the preferred location of most drug-metabolizing P450 isoenzymes. These findings are compatible with a metabolically mediated hepatocellular liver injury. A genetic polymorphism of any enzyme involved in drug detoxification could cause an accumulation of the parental drug or its metabolites, predisposing to anastrozole-induced liver toxicity. Liver function parameters rapidly improved after drug withdrawal in the reported cases.

## 9. Hepatotoxicity of Specific-Targeted Antibodies

### 9.1. Lapatinib-Induced Hepatitis

Lapatinib is an inhibitor of the tyrosine kinases of human epidermal growth factor receptor type 2 (HER2) and epidermal growth factor receptor type 1. A number of studies have shown that lapatinib has clinical activity in patients with HER2-positive breast cancer, with a significant reduction in the risk of disease progression [61]. In a phase II trial, grade 3 and 4 liver toxicity were uncommon after single agent lapatinib [61]. In one report [62] a women with advanced breast cancer who had been treated with lapatinib for 14 days developed severe hepatitis. Liver biopsy showed portal-to-portal and portal-to-central bridging necrosis, foci of severe hemorrhage, and hepatocellular dropout around the centrilobular areas. Eosinophil infiltrate was seen in many portal spaces. These findings are all consistent with drug-induced hepatitis. Bilirubin and liver enzymes returned to normal within three months of lapatinib discontinuation. A recent study [63] has identified and confirmed associations between lapatinib-associated liver injury and the highly correlated MHC class II alleles *HLA-DQA1∗02:01*, *DRB1∗07:01*, and *DQB1∗02:02* plus an SNP in the same genomic locus, *TNXB *(rs12153855).

### 9.2. Inflammatory Hepatotoxicity of CTLA-4 Antibody Therapy

The cytotoxic T-lymphocyte (CTLA-4) receptor binds molecules of the B7-family which leads to a suppression of T cells. Specific CTLA-4 antibodies induce an unrestrained T-cell activation. Treatment with the CTLA-4 antibodies ipilimumab and tremelimumab has been approved for metastatic melanoma [64]. A unique set of adverse effects may occur, termed immune-related adverse events. These include rashes and colitis, usually mild to moderate. Less frequent manifestations such as, hypophysitis, hepatitis, pancreatitis, iridocyclitis, lymphadenopathy, neuropathies, and nephritis have also been reported [64]. Immune-related hepatotoxicity was observed in 3% to 9% of patients receiving anti-CTLA-4 antibodies [65, 66] manifested as an asymptomatic increase of aminotransferases and bilirubin, although some patients also had fevers and malaise. A waxing and waning picture may be seen. Biopsies showed a diffuse T-cell infiltrate consistent with immune-related hepatitis. It has been recommended that for grades 3 to 4 hepatotoxicity, one should use high-dose intravenous glucocorticosteroids. If the condition persists, immunosuppressant therapy with mycophenolate mofetil may also be considered. Infliximab, because of its potential for hepatotoxicity, should be avoided in this setting [64]. 

## 10. Radiation-Induced Liver Disease

### 10.1. Liver Injury from External Beam Radiation

Radiation induced liver disease (RILD) after conventionally fractionated radiotherapy was first described several decades ago, and it was soon thereafter recognized to have the histopathologic features of sinusoidal of venoocclusive disease (VOD), currently termed sinusoid obstructive syndrome (SOS) [67, 68]. The clinical scenario commonly called “classic” radiation induced liver disease (RILD) occurs typically within 4 months after hepatic radiation therapy. It is characterized by anicteric ascites and hepatomegaly and an isolated elevation in alkaline phosphatase disproportionate to that of other liver enzymes. “Classic” RILD is unlikely to occur after a mean liver dose of approximately 30 Gy in conventional fractionation. Patients with underlying chronic hepatic disease may present with liver function abnormalities that do not match the criteria described previously, including jaundice and/or Markedly elevated serum transaminases (more than 5 times the upper limit of normal) within 3 months of completion of hepatic radiation therapy [69–71]. All these hepatic toxicities have been included under the umbrella label of “nonclassic RILD.” It was postulated that radiation injury to sinusoidal endothelial cells and central vein endothelium initiates activation of the coagulation cascade, leading to accumulation of fibrin and formation of clots in the central veins and hepatic sinusoids [72]. The ensuing hypoxic milieu presumably results in the death of centrilobular hepatocytes and atrophy of the inner hepatic plate, producing the hepatic dysfunction. By maintaining a low mean liver dose and sparing a “critical volume” of liver from radiation, stereotactic delivery techniques allow for the safe administration of higher tumor doses. Caution must be exercised for patients with hepatocellular carcinoma or preexisting liver disease (e.g., Child-Pugh score of B or C) because they are more susceptible to RILD that can manifest in a nonclassic pattern. No pharmacologic interventions have yet been proven to mitigate or treat RILD. 

### 10.2. Radioembolization-Induced Liver Injury

Selective internal radiation therapy (SIRT) [73] with 90yttrium microspheres is a relatively new clinical modality for treating nonresectable malignant liver tumors. This interventional radiology technique employs percutaneous microcatheterisation of the hepatic arterial vasculature to selectively deliver radioembolic microspheres into neoplastic tissue. SIRT results in measurable tumor responses or delayed disease progression in the majority of eligible patients with hepatocellular carcinoma or hepatic metastases arising from CRC. It has also been successfully used as palliative therapy for noncolorectal malignancies metastatic to the liver. Side effects are not common after radioembolization. A postembolization syndrome like the one that appears after TACE is not seen. Radioembolization is safe in patients with portal vein thrombosis [74] in whom TACE may lead to complications such as liver abscess or decompensation of cirrhosis [75]. Results from a small series suggest that it could also be safe in asymptomatic patients with lobar or segmental biliary tract obstruction but normal or near-normal bilirubin [76]. Safety in these special situations is probably the result of the lack of significant ischemia [77]. However, radioembolization may produce relevant toxic effects as a result of radiation of nontarget organs including, cholecystitis, gastrointestinal ulceration, pneumonitis, and most importantly for HCC patients, liver toxicity. Two consequences of cirrhosis may affect radioembolization in the cirrhotic liver. On one hand, the usual distribution of microspheres can be profoundly altered by the vascular changes and the presence of anatomical arterioportal and arteriovenous shunts. This may modify the radiation dose absorbed by the tumor and the nontumoral liver and therefore affect treatment tolerance and effectiveness. On the other hand, the cirrhotic liver has a reduced functional reserve that produces an increased risk of liver failure after liver insults including external irradiation [78]. A direct liver cell injury and a further compromise of liver blood supply produced by radiation-mediated blood vessel damage could all result in a higher risk of clinically relevant liver toxicity after radioembolization in comparison with noncirrhotic livers. Microspheres are often distributed in a heterogeneous way and not infrequently form clusters. This may explain the lack of a clear dose-event relationship in liver tolerance, as happens with tumor response. The general agreement is that the dose absorbed by the nontumoral liver tissue should be kept below 50 Gy for patients with cirrhosis [79]. In non-cirrhotic patients, a form of sinusoidal obstruction syndrome appearing 4–8 weeks after radioembolization as jaundice, mild ascites, and a moderate increase in GGTP and alkaline phosphatase has been described as radioembolization-induced liver disease (REILD) [80]. The actual incidence of this complication in cirrhotics and noncirrhotics is difficult to establish because most published series report on changes in individual laboratory values along different periods of time. In those populations with a predominance of cirrhotic patients, abnormal liver function tests are frequently present at baseline and liver failure may develop as a result of the progression of the chronic liver disease. Thus, differences in reporting criteria may disturb the estimation of the incidence of REILD. In the two largest series ever published [81, 82], grade 3 or higher CTCAE bilirubin levels (a hallmark of REILD) were observed within 3 months after therapy in 14% and 6% of patients treated with glass or resin spheres, respectively. Although a causal relationship with radioembolization could only be confirmed in a controlled clinical, it is very likely that the increased bilirubin levels reflect some kind of REILD. 

## 11. Summary and Outlook

Liver injury secondary to cytotoxic chemotherapy as well as novel molecular targeted and biological agents is one of the most serious adverse effects of anticancer treatment. Liver damage can assume diverse clinical and histological forms that are specific to the offending agent for most cases. Not only can hepatotoxicity culminate in liver failure and death, but it may also result in a postponement of scheduled treatment or complicate hepatic resection with curative intent. Our knowledge about the deleterious effects of chemotherapeutic drugs and innovative radiation treatment is far from complete. We need to fill this gap by several methods, for example, employing basic laboratory methods on animal models of liver injury induced by chemotherapy and multicenter registration of all cases of hepatotoxicity that include detailed clinical and laboratory data for each case. Finally, we have to initiate studies on modes to prevent or mitigate liver injury. Hepatologists and oncologists have to cooperate closely in this underappreciated topic. 
